# Transcriptional regulation of kinases downstream of the T cell receptor: another immunomodulatory mechanism of glucocorticoids

**DOI:** 10.1186/2050-6511-15-35

**Published:** 2014-07-03

**Authors:** Maria Grazia Petrillo, Katia Fettucciari, Paolo Montuschi, Simona Ronchetti, Luigi Cari, Graziella Migliorati, Emanuela Mazzon, Oxana Bereshchenko, Stefano Bruscoli, Giuseppe Nocentini, Carlo Riccardi

**Affiliations:** 1 Department of Medicine, University of Perugia, Perugia, Italy; 2 Department of Experimental Medicine, University of Perugia, Perugia, Italy; 3 Department of Pharmacology, Faculty of Medicine, Catholic University of the Sacred Heart, Rome, Italy; 4 Istituto di Ricovero e Cura a Carattere Scientifico (IRCCS) Centro Neurolesi “Bonino-Pulejo”, Messina, Italy; 5 Department of Medicine, Section of Pharmacology, Severi Square 1, University of Perugia, I-06132 San Sisto, Perugia, Italy

**Keywords:** Glucocorticoids, Gene modulation, Kinases, In vivo treatment, T cells, Thymocytes, Apoptosis, Knock-out mice

## Abstract

**Background:**

Glucocorticoids affect peripheral immune responses, including modulation of T-cell activation, differentiation, and apoptosis. The quantity and quality of T-cell receptor (TCR)-triggered intracellular signals modulate T-cell function. Thus, glucocorticoids may affect T cells by interfering with the TCR signaling cascade. The purpose of the study was to search for glucocorticoid-modulated kinases downstream of the TCR.

**Methods:**

Gene modulation in lymphoid cells either treated with glucocorticoids or from glucocorticoid-treated mice was studied using a RNase protection assay, real-time PCR, and western blotting. The sensitivity of genetically modified thymocytes to glucocorticoid-induced apoptosis was studied by performing hypotonic propidium iodide staining and flow cytometry. The Student’s t-test was employed for statistical evaluation.

**Results:**

We found that transcription of Itk, a non-receptor tyrosine kinase of the Tec family, was up-regulated in a mouse T-cell hybridoma by the synthetic glucocorticoid dexamethasone. In contrast, dexamethasone down-regulated the expression of Txk, a Tec kinase that functions redundantly with Itk, and Lck, the Src kinase immediately downstream of the TCR. We investigated the expression of *Itk*, *Txk*, and *Lck* in thymocytes and mature lymphocytes following in vitro and in vivo dexamethasone treatment at different time points and doses. Kinase expression was differentially modulated and followed distinct kinetics. *Itk* was up-regulated in all cell types and conditions tested. *Txk* was strongly up-regulated in mature lymphocytes but only weakly up-regulated or non-modulated in thymocytes in vitro or in vivo, respectively. Conversely, *Lck* was down-regulated in thymocytes, but not modulated or up-regulated in mature lymphocytes in the different experimental conditions. This complex behaviour correlates with the presence of both positive and negative glucocorticoid responsive elements (GRE and nGRE, respectively) in the *Itk, Txk and Lck* genes. To investigate the function associated with *Itk* up-regulation, dexamethasone-induced apoptosis of thymocytes from *Itk*-deficient mice was evaluated. Our results demonstrated that *Itk* deficiency causes increased sensitivity to dexamethasone but not to other pro-apoptotic stimuli.

**Conclusions:**

Modulation of *Itk*, *Txk*, and *Lck* in thymocytes and mature lymphocytes is another mechanism by which glucocorticoids modulate T-cell activation and differentiation. *Itk* up-regulation plays a protective role in dexamethasone-treated thymocytes.

## Background

Glucocorticoids are used to treat several autoimmune diseases and prevent organ rejection following transplantation due to their potent anti-inflammatory and immunosuppressive activity. These compounds can also inhibit lymphocyte proliferation and induce lymphocyte death [1-3]. However, glucocorticoids have also been reported to potentiate the immune response and modulate lymphocyte differentiation. For example, when used in some experimental systems, these compounds stimulate T-cell receptor (TCR)–mediated T-cell proliferation and inhibit activation-induced cell death [4]. At physiologic concentrations, glucocorticoids shift immunity from a T helper (Th)1 to a Th2 response, and promote production of T regulatory (Treg) cells [5-7]. This shift is also observed following long-term treatment with glucocorticoids [8].

Recognition of the antigen-major histocompatibility complex (MHC) by the TCR leads to a cascade of signaling events initiated by activation of lymphocyte protein tyrosine kinase (Lck), a Src-family kinase crucial in T-cell development and activation [9]. Lck phosphorylates the cytoplasmic domain of CD3, leading to activation of ZAP-70, LAT, and SLP-76, which in turn serve as a platform for recruiting molecules, such as Tec kinases, into the signalosome [10]. Among Tec kinases, IL-2 inducible T cell kinase (Itk) is expressed most highly and exerts the greatest effects on T-cell function [11,12]. Txk tyrosine kinase (Txk) is a Tec kinase that exhibits partial redundancy with Itk and is expressed at lower levels than Itk [12]. Mice deficient in *Itk* (*Itk*^*-/-*^) show altered T-cell development and impaired T-cell effector function. Deletion of both *Itk* and *Txk* causes marked defects in TCR responses, including proliferation, cytokine production, and apoptosis in vitro, as well as a dysfunctional immune response to *Toxoplasma gondii* infection in vivo [13]. Molecular events immediately downstream of the TCR are intact in *Txk*^*-/-*^*Itk*^*-/-*^ cells; however, intermediate events such as inositol triphosphate production, calcium mobilization, and mitogen-activated protein kinase activation are impaired. These data establish Tec kinases as critical regulators of TCR signaling required for phospholipase C-γ activation [13].

Other data suggest that Itk and Txk play a divergent role in T cell differentiation. *Itk*^*-/-*^ mice are unable to mount a Th2 response in models of allergic asthma [14], as well as following infection with *Leishmania major*, *Nippostrongylus brasiliensis*, and *Schistosoma Mansoni*, which lead to Th1 cytokine production [15,16]. Surprisingly *Itk*^*-/-*^*Txk*^*-/-*^ mice mount Th2 responses, which possibly suggests that these Tec kinases are involved in Th1/Th2 polarization. Indeed, Txk over-expression has been found to increase IFN-γ production. Moreover, increased Txk expression has been observed in patients with Behcet’s disease, a disorder associated with increased inflammation and Th1 cytokine production [17].

In this study, we demonstrate that the synthetic glucocorticoid dexamethasone modulates the expression of *Lck*, *Itk*, and *Txk* in thymocytes and mature lymphocytes in vitro and in vivo. In addition, we report that *Itk* up-regulation in thymocytes has a functional role, as demonstrated by the increased sensitivity of *Itk*^*-/-*^ thymocytes to dexamethasone-induced apoptosis. Thus, our data suggest that glucocorticoids modulate T-cell function and the immune response by fine-tuning the expression of Lck and Tec kinases.

## Methods

### Animals

C3H and BALB/c mice were purchased from Jackson Laboratory (Maine, USA). Itk^-/-^ BALB/c mice were a kind gift of dr. Locksley [15]. The animals were housed in a controlled environment, provided with standard rodent chow and water ad libitum and kept under specific pathogen-free conditions. Animal care was in compliance with regulation in Italy (Decreto Ministeriale 116192), Europe (Official Journal of European Contract Law 358/1 12/18/1986) and USA (Animal Welfare Assurance No A5594-01, Department of Health and Human Services, USA). The study was approved by the Italian Ministero della Salute.

For in vivo experiments, female mice were injected with 0.2 ml of saline solution or 5/25 mg/Kg dexamethasone in 0.2 ml saline solution and sacrificed 3 or 6 h after treatment.

### Cell isolation and treatment

Spontaneously dividing CD3^+^, CD4^+^, CD2^+^, CD44^+^ cells of the OVA-specific hybridoma T-cell line 3DO [18] obtained by recloning the original line in our laboratory were used. Cells were maintained in logarithmic growth at 37°C, 5% CO_2_ in RPMI 1640 medium supplemented with fetal bovine serum (10%, FBS), 10 mM Hepes and antibiotics and were treated with 10^-7^ M dexametasone for 24 h.

Thymuses, spleens and cervical, brachial, axillary, superficial inguinal and mesenteric lymph nodes were isolated from 4- to 5-week old female C3H (in vitro/vivo gene modulation experiments) or female BALB/c (apoptosis experiments) mice, teased in RPMI 1640 medium and directly processed (in vivo experiment) or resuspended in RPMI 1640 supplemented with fetal bovine serum (10%, FBS), 10 mM Hepes and antibiotics (in vitro experiments). In treated groups, dexamethasone was added for 1 and 3 h (RPA experiments and real-time PCR, 10^-7^ M) or 18 h (apoptosis experiments, concentration 2.5×10^-8^-10^-7^ M).

Total, CD4^+^ and CD8^+^ T lymphocytes were obtained from spleen. Briefly, after red blood cell lysis, single cell suspension was incubated either with CD4 plus CD8 microbeads (Miltenyi Biotec, Bergisch Gladbach, Germany) to get T lymphocytes or separately to obtain CD4^+^ and CD8^+^ cells. Magnetic retained cells where then eluted from LS columns according to the manufacturer’s instructions. The purified cells were shown to be >98% CD3^+^, CD4^+^ or CD8^+^ cells by flow cytometry.

### Promoter analysis

Putative glucocorticoid responsive element (GRE) and negative GRE (nGRE) sites within the murine regulatory regions of Itk, Txk and Lck genes were analyzed by MatInspector software algorithms (Genomatix). The core similarity was set to 75%, while matrix similarity was set to ≥0.85 (a perfect match to the matrix gets a score of 1).

### Differential display and cloning

RNA from untreated and dexamethasone-treated cells was isolated using Trizol LS reagent (Ambion, Life Technologies) according to the manufacturer’s instructions. Briefly, 750 μl TRIzol LS was added to 250 μl medium containing 20–40 × 10^6^ cells. After centrifugation with chloroform, RNA was precipitated by isopropanol and then washed with 75% ethanol.

DNA-free RNA (0.1 μg) was retrotranscribed (Moloney murine leukemia virus reverse transcriptase from Invitrogen, Life Technologies) with an anchored primer (T_11_AC) and forty cycles of PCR were performed by using T_11_AC and the OPA 5’-CGCGGAGGTG-3’ [19]. Three independent samples of untreated 3DO cells were compared to three samples of 24 h dexamethasone-treated 3DO cells, by SDS-polyacrylamide gel. The radioactive band present in treated samples yet absent in the untreated cells was extracted, cloned and sequenced.

### PCR and RACE

RNA was purified and retrotranscribed as above specified but poly-T primer was used. To exclude any contamination from cellular DNA, DNAse-treated RNA was also used. Itk intron 5 was amplified by PCR using the primers 5’-TGGGCTGTGTCTATTCCCTGCCATG-3’ and 5’-TAGAATTGTGGAGCTGAACAG-3’ and using cDNA from RNA or DNA-free RNA. In order to further exclude that Itk intron was amplified from DNA, PCR was also performed with RNA that was not retrotranscribed. 5’ rapid amplification of cDNA ends (RACE) was performed as previously described [20].

### RNAse protection assay (RPA)

RNA was isolated as above specified and RPA was performed as previously specified [21]. Probes for RNase protection were constructed by RT-PCR using primers 5’-GCTTGGTGCATATCCTTCATG-3’ and 5’-CGGTCATTTCAGGAACCTGAAG-3’ for Itk, 5’-AAAACATTCCCAGCGTCAGAGG-3’ and 5’-GCAGCGGCTTGCGCTTCGAAGG-3’ for Txk and 5’-GAGCAGAGCGGTGAGTGGTGG-3’ and 5’-TGCCGCTCGGCGTCCTTACGG-3’ for Lck and inserted in the pCRII vector (Invitrogen, Life Technologies). pCRII-Itk, pCRII-Txk and pCRII-Lck RNA probes were 301, 215, 273 bp long and protected a fragment of 174 (encompassing exon 6 and 7), 88 (encompassing exon 3 and 4), 146 bp (encompassing exon 4–6), respectively. Plasmids were linearized with BamHI (New England Biolabs). The probe giving a 250-bp fragment (β-actin probe) protecting β-actin was purchased from Ambion (Life Technologies), linearized with XbaI, and used as internal control. All the probes were transcribed with T7 RNA polymerase (Ambion, Life Technologies) in the presence of 5 μCi [α-^32^P]UTP; β-actin was transcribed in the presence of 0.5 μCi [α-^32^P]UTP. After washing out unincorporated nucleotides by quick spin columns (Fine Sephadex G-50, GE Healthcare, Life Sciences), 5 × 10^5^ cpm probe (5 × 10^4^ cpm β-actin) was hybridized to total cell RNA (10 μg) and then incubated overnight at 60°C.

RNase digestion was performed by using an RNase A (Roche)(40 μg/ml) and an RNase T1 (Ambion, Life Technologies)(1.5 U/μl) solution at 20°C for 5 min. The undigested products were treated with phenol-chloroform, precipitated with ethanol, and loaded on a denaturing polyacrylamide sequencing gel. The gel was exposed to Biomax MR Film Kodak (Sigma-Aldrich) with intensifying screens at -70°C for 12 h to 2 days to obtain images of good quality.

Quantitation of protected bands was obtained evaluating cpm by Imager-Packard [22]. The ratio cpm of Itk, Txk or Lck protected fragment/cpm of β-actin protected fragment was calculated for each RNA and modulation of gene expression was equal to the ratio “value dexamethasone-treated cells”/“value medium-treated cells” or “value cells from dexamethasone-treated animals”/“value from saline solution-treated animals” or “value from untreated animals”, as specified. In vitro and in vivo experiments were performed three times. Cells of in vivo experiments were pooled from 3–4 animals of the same experimental group.

### Real time PCR

RNA was purified as above specified. Conversion of total RNA (1 μg) to cDNA was performed with QuantiTect Reverse Transcription protocol (Qiagen).

Real time PCR was done with a 7300 Real time PCR system (Applied Biosystem) real time cycler using specific FAM/MGB dye-labeled TaqMan probes: Itk (Mm 00439862_m1), Txk (Mm 01213032_m1), Lck (Mm 00802897_m1). Gene expression was quantitated relatively to the expression of endogenous control mouse beta-actin (4352341e) VIC/MGB probe amplified in the same tube of investigated genes. All probes were purchased from Applied Biosystem. All experiments were carried out in triplicate and the ΔΔ^Ct^ method was used to determine expression of the genes of interest, as previously described [23].

### Western blotting

Cells were washed and lysed in SDS sample buffer for 30 min on ice. After centrifuging, the cleared lysates were boiled and run in a 10% SDS-polyacrylamide gel. The proteins were then transferred onto nitrocellulose membranes, which were hybridized with rabbit anti-Itk (Upstate, Merck-Millipore) antibody (Ab). Immunoreactive protein bands were visualized using horseradish peroxidase-conjugated goat anti-rabbit IgG (Pierce, Thermo Scientific) followed by enhanced chemioluminescence (Merck-Millipore).

Western blot plates were scanned and band signal intensities were determined using ImageJ software. Expression levels were normalized to β-tubulin (Sigma-Aldrich) expression.

### Apoptosis

Cells were treated with 10^-7^ M dexamethasone and plastic-coated anti-CD3 Ab (0.5 μg/ml) (Pharmingen) [24]. To evaluate heath shock-induced apoptosis, cells were kept at 43°C for 10, 20 or 30 min. Before evaluating the apoptosis levels by ipothonic propidium iodide staining [25], cells were cultured at 37°C, 5% CO_2_ for 18 h. Flow cytometric analysis was conducted on a Beckman Coulter EPICS XL-MCL running EXPO32 ADC analysis software.

### Statistical and mathematical analysis

Results were normally distributed and are the mean ± SD. Student’s t-test was adopted for statistical evaluation (*P < 0.05, **P < 0.01, ***P < 0.001). In apoptosis experiments, apoptosis caused by the treatment (specific apoptosis percentage) was calculated as follows: 100×[% apoptosis (treated cells)-% apoptosis (medium treated cells)]/[100-% apoptosis (medium treated cells)].

## Results

### Dexamethasone up-regulates *Itk* expression but down-regulates *Txk* and *Lck* in 3DO cells

**Figure 1:**
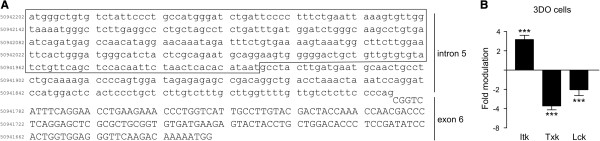
***Itk*, *Txk*, and *Lck *gene expression is modulated by dexamethasone in the T cell hybridoma 3DO. (A)** The DNA amplified and isolated by the differential display technique (included in the box) is a 275-bp fragment belonging to intron 5 of the *Itk* gene in proximity to exon 6. In the original fragment, the first and last 10 bp were identical/complimentary to the OPA (the primer used in differential display) used in the PCR reaction and overlapped only partially with the sequence of intron 5 (data not shown). **(B)***Itk*, *Txk*, and *Lck* expression in 3DO cells is modulated by 24-h treatment with dexamethasone (100 nM) as demonstrated by the RNAse protection assay. Fold modulation was calculated by dividing the *Itk*/β-actin cpm ratio of DEX-treated cells by that of the respective untreated control. Values represent the mean (±SD) of three independent experiments. ***P < 0.001, according to Student’s t-test comparing the levels of kinase mRNA in dexamethasone-treated cells to those in medium-treated control cells.

We performed differential display to investigate Tec kinase gene expression in 3DO T cells that were untreated or treated for 24 h with the synthetic glucocorticoid dexamethasone. A 275-bp cDNA segment was amplified at much higher levels in dexamethasone-treated cells compared to untreated cells. Upon sequencing and alignment, this segment was identified as belonging to the fifth intron of the *Itk* gene (Figure 1A). Because this segment was not amplified in non-transcribed samples, we concluded that the segment was derived from an RNA sequence that was retrotranscribed into cDNA. However, NCBI databases do not contain EST sequences and our attempts to clone a full-length cDNA containing an open reading frame by rapid amplification of cDNA ends (RACE) were unsuccessful. The only PCR product that we obtained was a longer segment belonging to intron 5 of *Itk*. The reason why an mRNA from an intron was transcribed by reverse transcriptase and amplified by differential display, PCR, and RACE remains unclear and is under investigation. One possibility is that this region has regulatory functions or results from pre-mRNA splicing, which is highly up-regulated by glucocorticoids.

We reasoned that if transcription of the intron is up-regulated, *Itk* transcription should also be up-regulated. Indeed, RNAse protection assay (RPA) confirmed our hypothesis because *Itk* transcription was up-regulated 4-fold in 3DO cells (Figure 1B). Considering that Itk is a kinase involved in TCR signaling, we investigated the effect of dexamethasone on the expression of other kinases activated by TCR stimulation, focusing our attention on Txk, the other relevant Tec kinase in T cells, and on Lck. In 3DO cells, expression of *Lck* and *Txk* was down-regulated by dexamethasone (Figure 1B), thereby suggesting that Itk, Txk, and Lck may play different roles in glucocorticoid-treated lymphocytes.

### Dexamethasone in vitro treatment up-regulates the expression of *Itk* and *Txk*, but not *Lck*, in thymocytes and peripheral T lymphocytes

**Figure 2:**
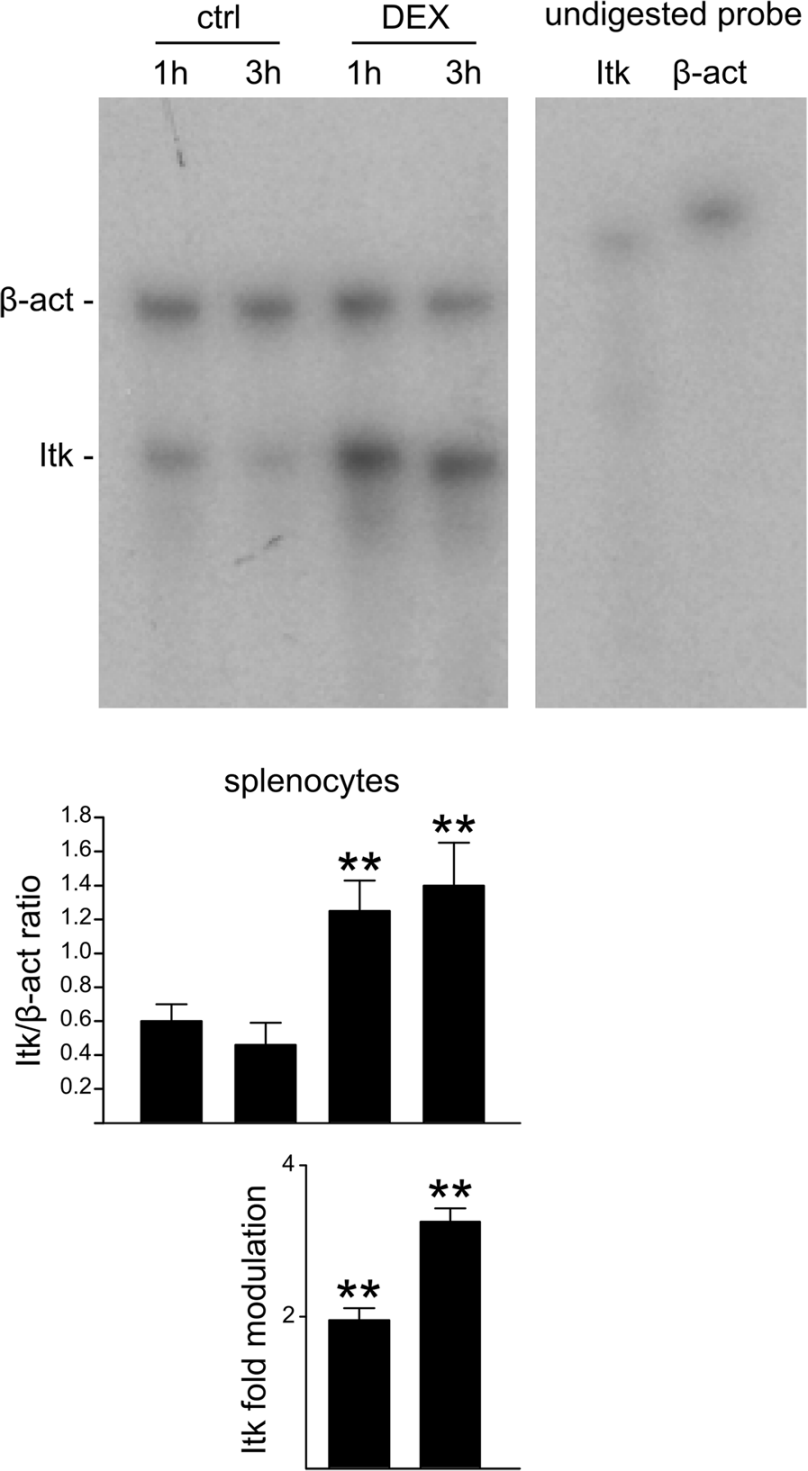
***Itk *gene expression is modulated in splenocytes.** RNAse protection assay was performed to evaluate the level of *Itk* mRNA in splenocytes that were untreated (ctrl) or treated with dexamethasone (DEX, 100 nM). The *Itk* probe protects a 174-bp fragment and the β-actin probe protects a 250-bp fragment. A representative experiment is shown in the top panel. After autoradiographic exposure (shown in the figure), the radioactivity of protected probes was evaluated using Instant Imager-Packard. The *Itk*/β-actin cpm ratio is reported in the middle panel. Fold modulation (bottom panel) was calculated by dividing the *Itk*/β-actin cpm ratio of DEX-treated cells by that of the respective untreated control. Values represent the mean (±SD) of three independent experiments. Fold modulation, shown on a logarithmic scale base 2 (bottom panel), were statistically significant (**P < 0.01, according to the Student’s t-test).

**Figure 3:**
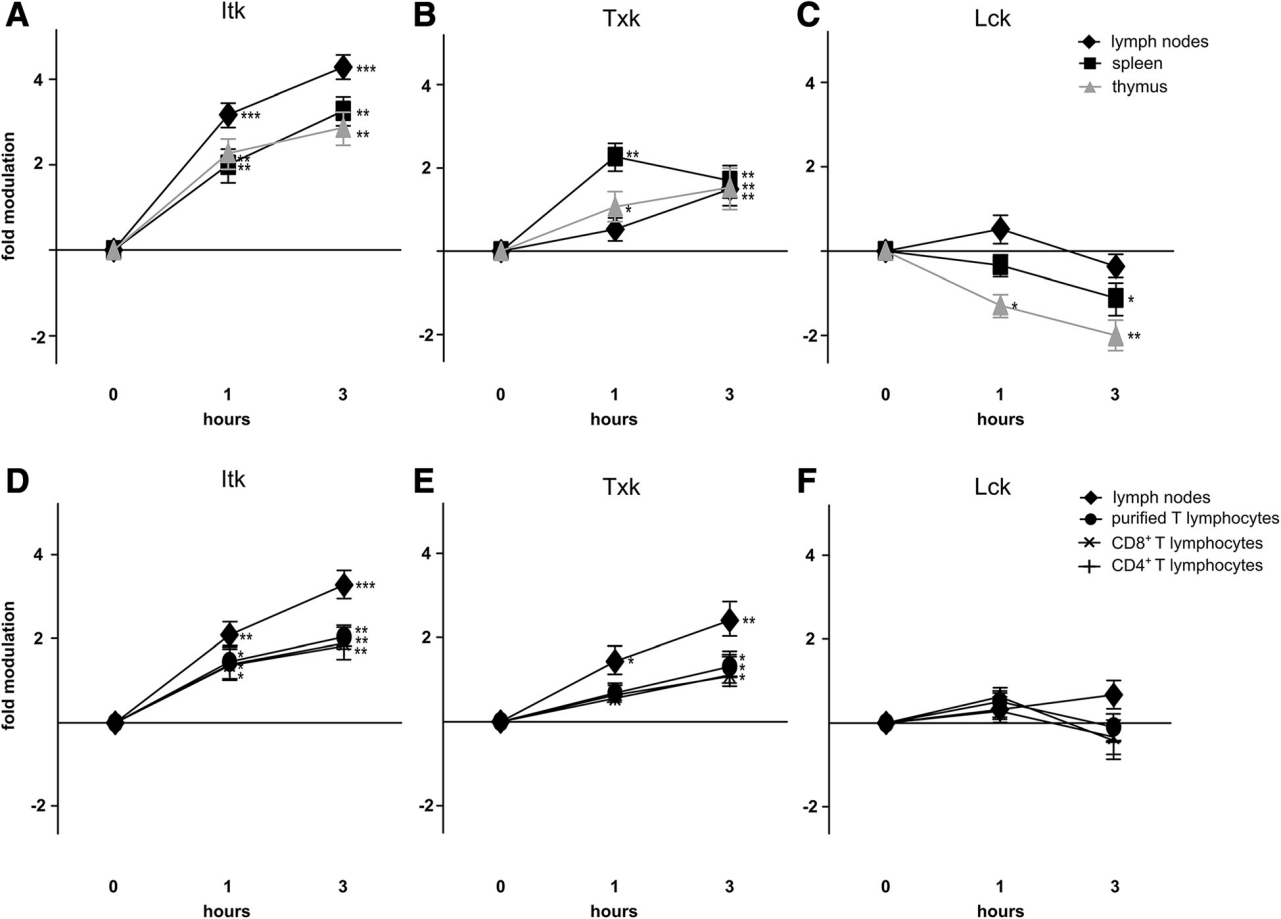
***Itk*, *Txk*, and *Lck *mRNA levels were modulated by dexamethasone treatment of immature and mature lymphocytes in vitro.***Itk*, *Txk*, and *Lck* mRNA levels were evaluated in thymocytes (gray triangle), splenocytes (black square), lymphocytes from lymph nodes (black diamond), peripheral T lymphocytes (circle), CD4^+^ T cells (plus), and CD8^+^ T cells (asterisk) that were untreated (0 h) or treated with medium alone or together with dexamethasone (DEX, 100 nM) for 1 or 3 h. Changes in mRNA expression were evaluated by RNAse protection assay and quantitated as shown in Figure 2 (panels A-C) or validated by real-time PCR (panel D-F). Fold modulation of *Itk***(A, D)**, *Txk***(B, E)**, and *Lck***(C, F)** expression is represented as the mean (±SD) of three independent experiments and is shown on a logarithmic scale (base 2). *P < 0.05, **P < 0.01, and ***P < 0.001, according to Student’s t-test comparing the levels of kinase mRNA in dexamethasone-treated cells to those in the respective controls.

Next, we examined the effect of dexamethasone on *Itk*, *Lck*, and *Txk* gene expression in murine thymocytes, splenocytes, and lymphocytes isolated from lymph nodes. For this, we used RPA because of its high sensitivity and accuracy in measuring mRNA levels [26,27]. For each probe, the cpm of the fragment protected by the gene under investigation was normalized to that protected by β-actin. Gene modulation was then quantified as the ratio of gene expression in treated versus untreated samples (Figure 2). Our data show that *Itk* expression increased in both thymocytes and peripheral T cells (Figure 3A), confirming the data obtained from dexamethasone-treated 3DO cells. Surprisingly, *Txk* expression also increased, albeit to a lower level than that of *Itk* (Figure 3B). Although dexamethasone significantly decreased *Lck* expression in thymocytes as previously demonstrated [28], little to no effect was observed in mature T-cell populations (Figure 3C).

Lck is expressed not only in T cells but also in NK cells, B cells, and dendritic cells (DCs) [28]. The modulation observed in spleen and lymph nodes may derive from modulation of Lck in both T and other cells. Itk and Txk are expressed almost exclusively in T and NK cells [12] but modulation observed in splenocytes and lymph node T cells may be due to indirect effects, such as interaction of T cells with other glucocorticoid-treated cells present in the culture (e.g., B cells). To confirm that the observed modulation was induced by dexamethasone treatment of T cells, we purified total, CD4^+^ and CD8^+^ T cells and evaluated the effect of glucocorticoid treatment by real-time PCR. The results confirmed that *Itk* and *Txk* expression in T cells increases over time similar to expression in lymph node cells from the same animals although the levels were lower (Figure 3D-E). *Lck* expression was not modulated in either T cells or lymph node cells (Figure 3F).

### *Itk*, *Txk*, and *Lck* are also modulated by dexamethasone treatment in vivo

**Figure 4:**
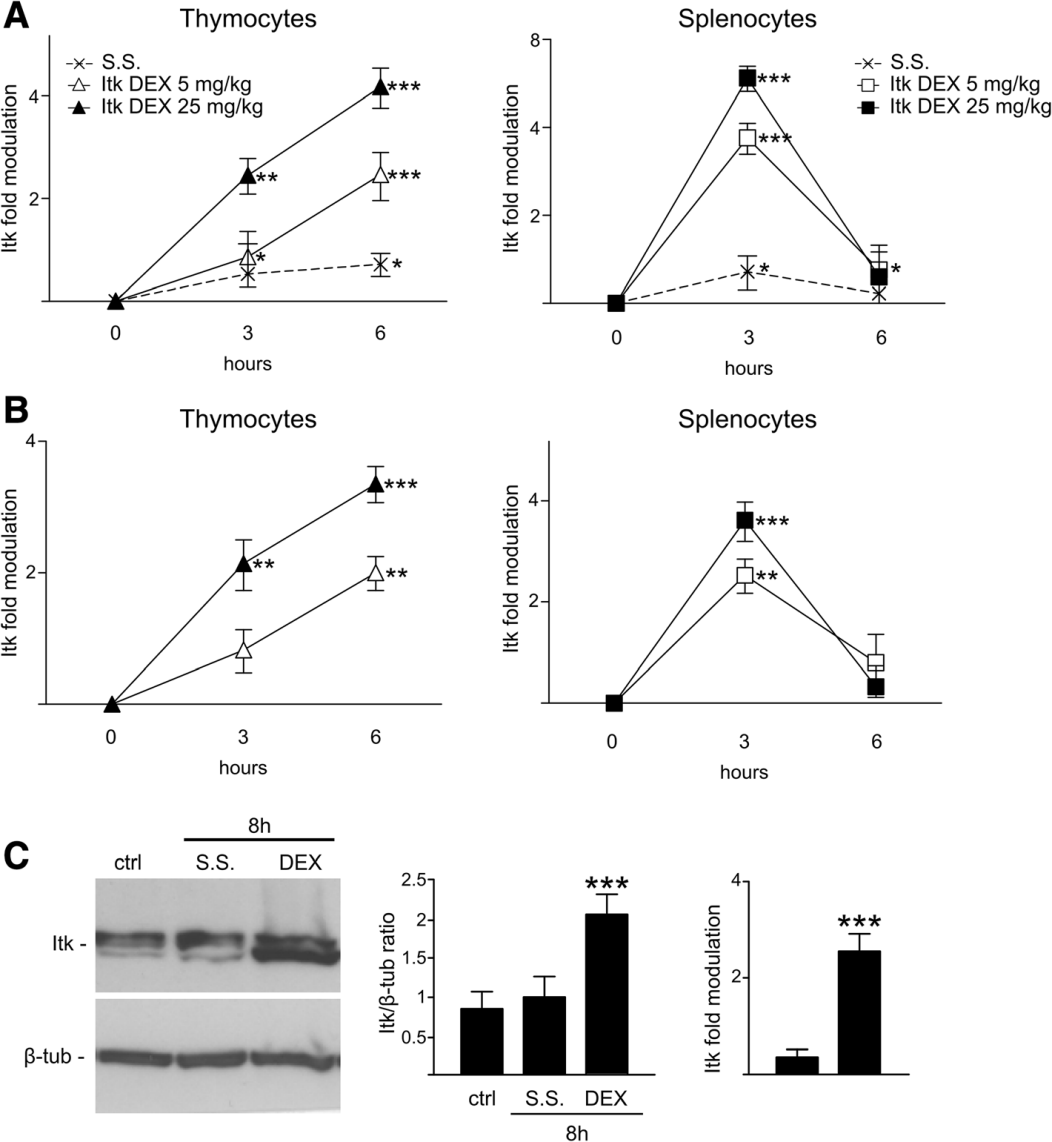
**Itk expression is modulated by dexamethasone treatment in thymocytes and splenocytes in vivo.** The level of *Itk* mRNA was evaluated in thymocytes (left panels) and splenocytes (right panels) isolated from mice treated with 5 mg/kg (empty symbol) or 25 mg/kg (black symbol) dexamethasone (DEX), or saline solution (S.S., asterisk). The animals were sacrificed 3 or 6 h after treatment. Changes in mRNA level were evaluated by RNA protection assay and evaluated as shown in Figure 2. Fold modulation is represented as the mean (±SD) of three independent experiments, with each experiment performed on cells pooled from three animals. The data are graphed on a logarithmic scale (base 2). *P < 0.05, **P < 0.01, and ***P < 0.001, according to Student’s t-test comparing the levels of *Itk* mRNA in cells from treated animals to that in cells from untreated animals **(A)** or from saline-treated animals **(B). (C)** Levels of Itk protein were evaluated in thymocytes from mice treated for 8 h with 5 mg/kg dexamethasone (DEX) or saline solution (S.S.). The left panel shows one representative experiment (n = 4). After densitometric analysis, the Itk/β-tubulin ratio (middle panel) and fold modulation (right panel) were calculated. ***P < 0.001, according to Student’s t-test comparing the level of Itk protein in thymocytes from DEX-treated animals to that in thymocytes from untreated and saline-treated animals.

We then proceeded to investigate whether dexamethasone affects the expression of these kinases in thymocytes and splenocytes following in vivo treatment. Mice were treated with either two doses of dexamethasone or with saline solution and then sacrificed after 3 or 6 h. The results show that *Itk* expression is significantly increased upon administration of both low and high doses of dexamethasone and that the increase is dose dependent (Figure 4). Although *Itk* expression in the thymus increased up to 6 h after administration, *Itk* expression in the spleen peaked at 3 h before returning to control levels after 6 h. Interestingly, despite possible differences between in vitro and in vivo treatments, including cellular redistribution, cross-talk, and pharmacokinetics, treatment with dexamethasone for 3 h modulated *Itk* expression similarly in vitro and in vivo. Of note, a slight but significant increase in *Itk* expression was also observed following saline treatment in both thymocytes and splenocytes (Figure 4A). This increase was most likely due to endogenous production of glucocorticoids following manipulation stress, supporting the idea that *Itk* modulation can occur with physiologic alteration of endogenous glucocorticoids. The observed up-regulation of *Itk* mRNA led to increased Itk protein levels as demonstrated by western blot analysis (Figure 4C).

**Figure 5:**
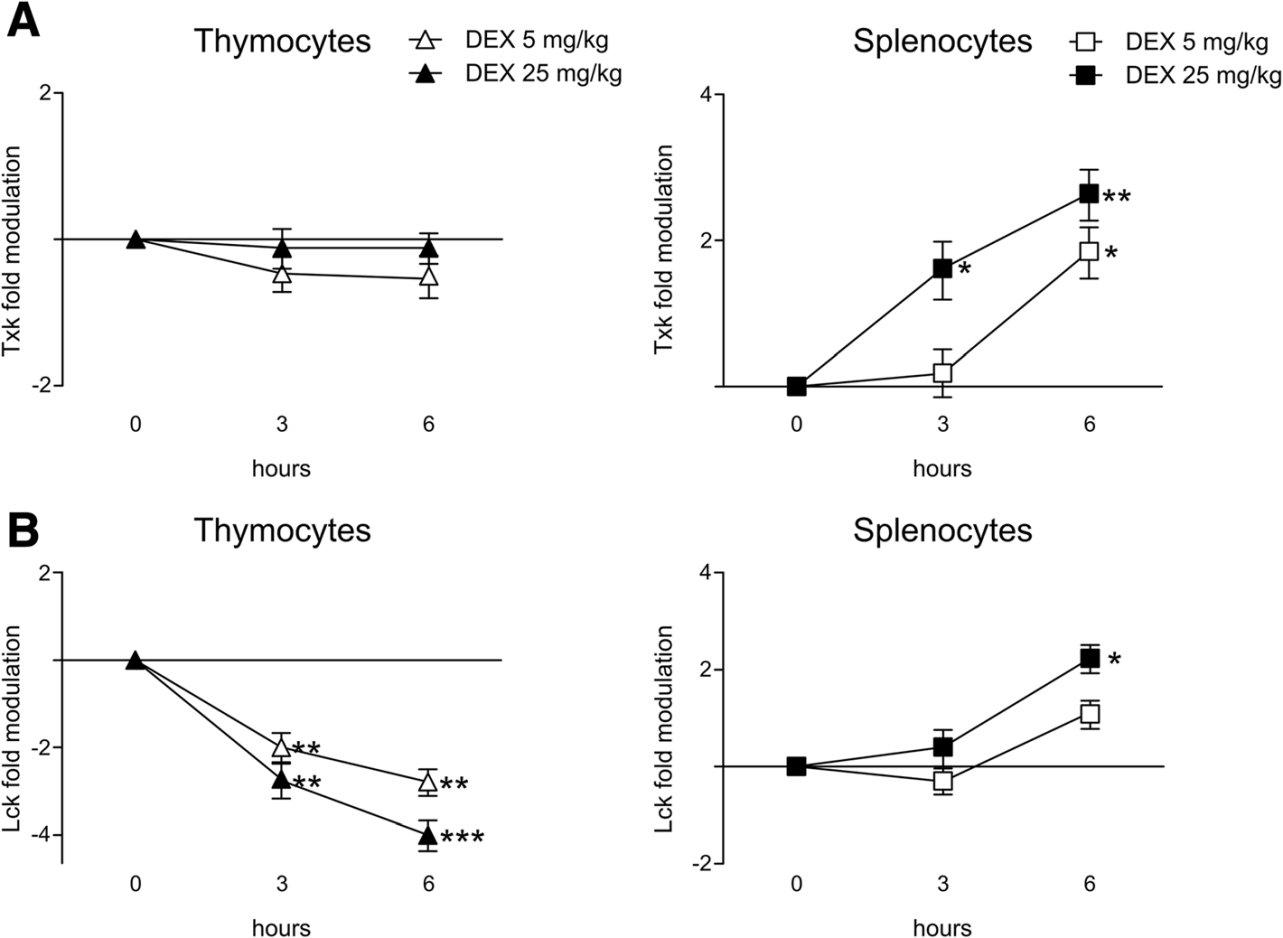
***Txk *and *Lck *gene expression is modulated by dexamethasone treatment of thymocytes and splenocytes in vivo.** Levels of *Txk***(A)** and *Lck***(B)** mRNA were evaluated in thymocytes (left panel) and splenocytes (right panel) from mice treated with 5 mg/kg (empty symbol) or 25 mg/kg (black symbol) dexamethasone, or saline solution. The animals were sacrificed 3 or 6 h after treatment. Changes in mRNA level were evaluated by RNAse protection assay as shown in Figure 2. Fold modulation is represented as the mean (±SD) of three independent experiments, with each experiment performed on cells pooled from three animals. The data are graphed on a logarithmic scale (base 2). *P < 0.05, **P < 0.01, and ***P < 0.001, according to Student’s t-test comparing the levels of kinase mRNA in cells from treated animals to that in cells from saline-treated animals.

Evaluation of glucocorticoid-induced in vivo modulation of the *Txk* gene revealed that, compared to saline-treated mice, gene expression was increased slightly in peripheral T cells after a 3 h treatment with the highest dexamethasone dose (Figure 5A, right panel), consistent with the reduced responsiveness to dexamethasone treatment observed in vitro. Both low and high dexamethasone doses were effective in promoting *Txk* modulation after treatment for 6 h. On the contrary, *Txk* expression did not change significantly in thymocytes following in vivo dexamethasone treatment (Figure 5A, left panel). Compared to saline-treated control animals, dexamethasone-treated mice exhibited strong down-regulation of *Lck* expression in the thymus (Figure 5B, left panel). This down-regulation was dose-dependent and consistent with results from the in vitro experiments. Comparison of saline-treated and untreated mice revealed significantly decreased *Lck* expression in saline-treated mice (1.7 fold, P < 0.01, data not shown). However, *Lck* was up-regulated significantly in mature lymphocytes just 6 h after administration of the highest dexamethasone dose (Figure 5B, right panel). Taken together, *Itk*, *Txk*, and *Lck* were modulated by dexamethasone treatment. Although these kinases are activated upon T-cell activation and participate in overlapping signaling pathways, dexamethasone modulated each kinase differently (summarized in Table 1).

**Table 1:** Summary of kinase modulation following dexamethasone treatment

		**Effect of dexamethasone treatment**		
		**In vitro 3 h treatment**	**In vivo 3 h treatment (25 mg/kg)**	**In vivo 6 h treatment (25 mg/kg)**
Thymocytes	*Itk*	↑↑↑	↑↑	↑↑↑
	*Txk*	↑	=	=
	*Lck*	↓↓	↓↓↓	↓↓↓
Lymphocytes from peripheral organs	*Itk*	↑↑↑	↑↑↑	=
	*Txk*	↑↑	↑↑	↑↑↑
	*Lck*	=	=	↑↑

=,gene expression difference between treated and medium-treated samples/animals is not significant;

↑ or ↓,The difference between treated and untreated samples is significant and has a fold modulation (increase or decrease) lower than 1.6 or higher than -1.6.

↑↑ or ↓↓,The difference between treated and untreated samples is significant and has a fold modulation comprised between 1.6 and 2.2 or -1.6 and -2.2.

↑↑↑ or ↓↓↓,The difference between treated and untreated samples is significant and has a fold modulation higher than 2.2 or lower than -2.2.

### Thymocytes from *Itk*^*-/-*^ mice were more sensitive to dexamethasone treatment

**Figure 6:**
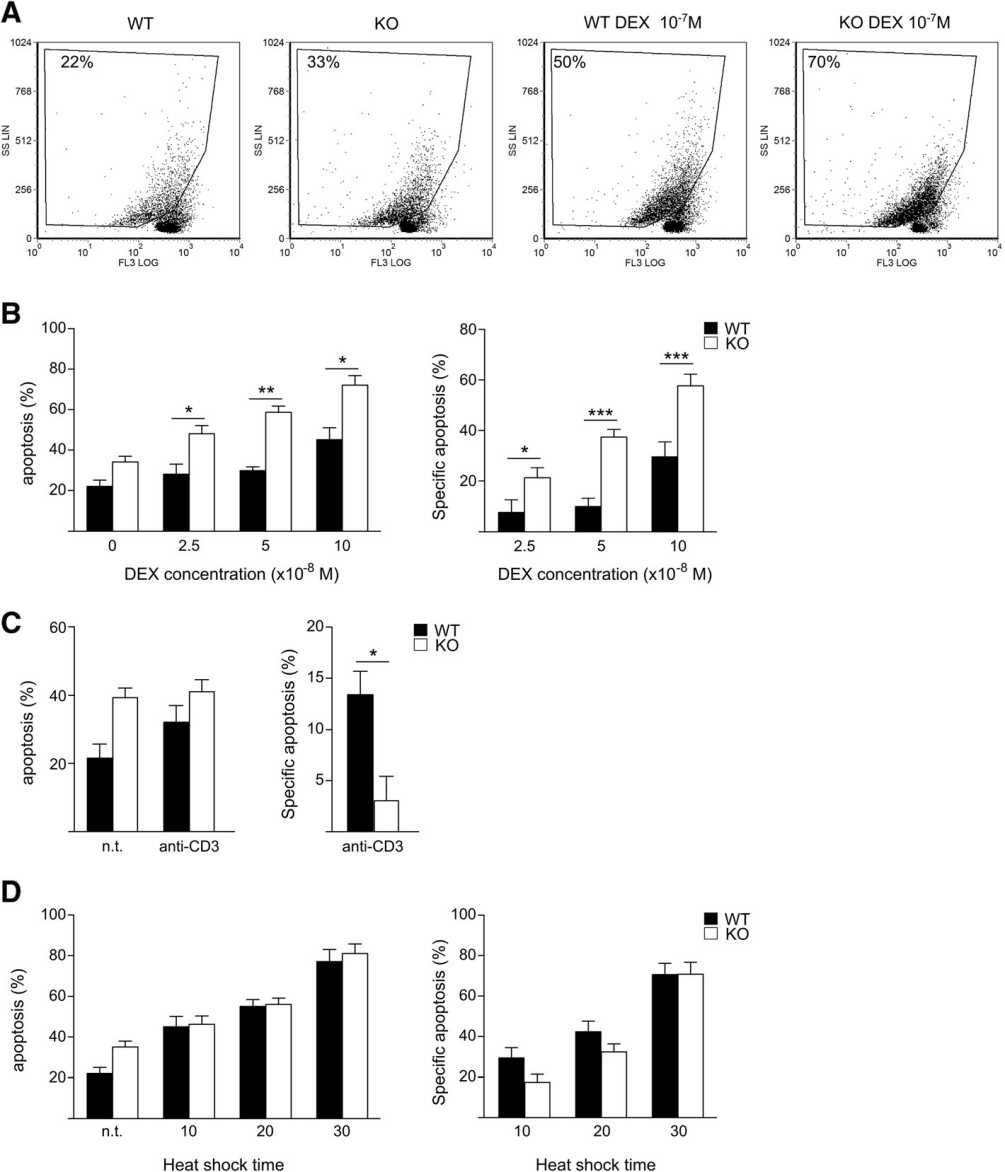
***Itk***^***-/- ***^**thymocytes are more sensitive than wild type thymocytes to apoptosis induced by dexamethasone but not by TCR activation or heat shock. (A)***Itk*^*-/-*^ (KO) and wild type (WT) thymocytes were left untreated or treated with 10^-7^ M dexamethasone (DEX) for 18 h before staining with a hypotonic solution of propidium iodide followed by flow cytometric analysis for apoptotic cells. One representative experiment performed on cells pooled from four mice is shown. The percentage of apoptotic cells within the gate is reported. **(B)** The percentage of apoptotic cells following treatment with the specified concentration of DEX is represented as the mean (±SD) of four independent experiments, performed on cells pooled from three to four mice. Because the level of spontaneous apoptosis was higher in KO than WT thymocytes, apoptosis in DEX-treated cells was normalized using the respective level of spontaneous apoptosis and the DEX-specific apoptosis was calculated as specified in Methods (right panel). **(C)** KO and WT thymocytes were treated with anti-CD3 antibody for 18 h and apoptosis was evaluated as above. The percentage of apoptotic cells is represented as the mean (±SD) of four independent experiments, with each experiment performed on cells pooled from three or four mice. Specific apoptosis is shown in the right panel. **(D)** KO and WT thymocytes were incubated at 43°C for the specified times and apoptosis was evaluated after a further 18 h as described above. The percentage of apoptotic cells is represented as the mean (±SD) of four independent experiments, with each experiment performed on cells pooled from three or four mice. Specific apoptosis is shown in the right panel. *P < 0.05, **P < 0.01, and ***P < 0.001, according to Student’s t-test comparing KO with WT apoptosis (left panel) or DEX-specific apoptosis (right panel).

To verify whether dexamethasone-induced Itk up-regulation plays a functional role, we treated *Itk*^*-/-*^ thymocytes with dexamethasone and evaluated apoptosis by flow cytometric analysis of propidium iodide-stained cells. Figure 6A and 6B (left panel) show that *Itk*^*-/-*^ thymocytes were more sensitive to dexamethasone-induced apoptosis than wild type thymocytes. A significantly higher percentage of apoptotic cells was observed even at the lowest dexamethasone concentration. At the same dexamethasone concentration (2.5 × 10^-8^ M), wild type thymocytes exhibited similar levels of apoptosis to that of untreated cells.

Interpretation of this data is complicated by the higher level of spontaneous apoptosis observed in *Itk*^*-/-*^ thymocytes compared to wild type thymocytes. To compare the levels of dexamethasone-induced apoptosis, we normalized the data against the levels of spontaneous apoptosis detected and then calculated dexamethasone-specific apoptosis (see Methods section for details). The differences in specific apoptosis between *Itk*^*-/-*^ and wild type thymocytes were significant at each tested concentration (Figure 6B).

To evaluate whether this increased dexamethasone-specific apoptosis in *Itk*^*-/-*^ thymocytes was caused by a greater susceptibility to apoptosis, we evaluated the effect of other pro-apoptotic stimuli. Our data demonstrate that *Itk*^*-/-*^ thymocytes were not more sensitive to apoptosis induced by anti-CD3 and heath shock (Figure 6C and 6D). Therefore, increased apoptosis in *Itk*^*-/-*^ thymocytes suggests that Itk plays a protective role in dexamethasone-induced apoptosis, which works to counteract this biological phenomenon.

## Discussion

In several cell types, including T cells, glucocorticoids modulate the transcription of hundreds of genes whose differential expression changes the fate and function of cells, causing apoptosis, maturation, and differentiation [1-3]. Here we demonstrate that dexamethasone modulates Lck and Tec kinases rapidly in thymocytes and/or mature lymphocytes. If we consider the pro-apoptotic role of dexamethasone in thymocytes, we would expect that genes with a pro-survival and activating function are down-regulated. Surprisingly, while *Lck* expression was found to be down-regulated, Tec kinase expression was up-regulated by dexamethasone. The regulation of Lck and Tec kinases was not coordinated even in mature lymphocytes, further suggesting that the effects of glucocorticoids in T cells, and specifically T-cell activation, are complex.

Our study clearly demonstrates for the first time that dexamethasone up-regulates *Itk* expression in thymocytes. In our previous study, we found that Itk was slightly up-regulated in CD4^+^CD8^+^ double positive thymocytes treated with dexamethasone for 3 h [23]. However, the up-regulation was not validated using other techniques and the increased expression appeared to be low (1.5-fold). The higher level of up-regulation observed in this study (i.e., greater than 2-fold) may be the result of different techniques and/or the presence of CD4^+^ and CD8^+^ single-positive thymocytes. The up-regulation was very rapid because a greater than 2-fold increase was observed after 1 h of dexamethasone treatment in vitro. Furthermore, these data are quantitatively relevant because an approximately 4-fold increase was observed after treatment for 6 h in vivo, where the tested concentration was about 10-fold higher than that tested in vitro. The up-regulation of *Itk* in thymocytes following in vivo treatment confirms the relevance of the in vitro data and demonstrates that it is present despite the thymic microenvironment, including TCR triggering and endogenous glucocorticoids. Itk up-regulation protects thymocytes from dexamethasone-induced apoptosis, as demonstrated by the specific increased sensitivity of *Itk*^*-/-*^ thymocytes to glucocorticoids along with their decreased/unchanged sensitivity to other pro-apoptotic stimuli. In this context, the increased level of *Itk* expression observed after in vivo treatment with a high dose of dexamethasone may be due, at least in part, to protection from dexamethasone-induced apoptosis for those thymocytes with high levels of *Itk* expression.

A protective function for up-regulation of prosurvival genes in cells undergoing apoptosis is well supported by the literature. For example, a previous study from our group demonstrated that 3-h dexamethasone treatment of CD4^+^CD8^+^ double-positive thymocytes resulted in transcriptional regulation of at least 10 genes with known protective functions [23], including interleukin-7 receptor, which delivers anti-apoptotic and activating signals to cells upon activation, and FKBP5 immunophilin, which inhibits the interaction between glucocorticoids and their receptor. Interleukin-7 receptor was also found to be up-regulated by glucocorticoids in mature T cells [29]. Moreover, FKBP5 has been reported to be up-regulated when endogenous glucocorticoid overproduction is stimulated [30].

Analysis of the presence of glucocorticoid responsive elements (GRE) revealed a very well-conserved GRE motif within intron 9 of the *Itk* gene (Additional file 1: Table S1 and Additional file 2: Figure S1) that may account for *Itk* up-regulation in thymocytes and peripheral T cells. Surprisingly, we also found a well-conserved negative GRE (nGRE) within the promoter region of *Itk*. Differences in the extent of *Itk* up-regulation and its kinetics in cell lines, thymocytes, splenocytes, lymphocytes from lymph nodes, and purified T lymphocytes may be due to the balanced effects of GRE and nGRE, transcription factors present in each cell type, basal level of expression of *Itk* in different cell types, and other signals modulating the effects of glucocorticoids. Interestingly, *Itk* up-regulation is higher in lymphocytes from lymph nodes than in splenocytes and T cells from splenocytes, suggesting that the microenvironment from which T cells are derived influences the effects of glucocorticoids.

Our data also reveal that *Lck* is down-regulated by dexamethasone in thymocytes. This effect was observed as early as 1 h after in vitro treatment and continued up to 6 h in vivo. To the best of our knowledge, *Lck* down-regulation has been reported by other studies but only following long treatment times. In rat thymocytes, a 24-h glucocorticoid treatment decreased Lck protein level [31]. Moreover, in murine thymocytes (from a different strain to the one used here), decreased *Lck* mRNA expression was observed after 12–24 h of glucocorticoid treatment [28]. Inhibition of this kinase by treatment with shRNA and the Src inhibitor dasatinib enhanced thymocyte sensitivity to dexamethasone [28], suggesting that Lck protects cells from glucocorticoid-induced apoptosis. Low levels of Lck were found in T cells from the spleen and lymph nodes of mice suffering from an experimental graft-versus-host reaction (GVHR), and *Lck* down-regulation was demonstrated to be dependent on glucocorticoids [32]. In the same model, the level of *Lck* in thymocytes remained unchanged with respect to the control. Taken together, our results (short-term treatment) are consistent with these reports (long-term treatment), and suggest that glucocorticoid-mediated *Lck* modulation is complex and time- and concentration-dependent.

Surprisingly, we did not find sequences with a match to nGRE matrixes greater than 0.85 (i.e., exhibiting a high probability of binding GR and inhibiting transcription) within the *Lck* promoter region. Instead, we found a sequence with a match to the GRE matrixes greater than 0.85 (Additional file 1: Table S1 and Additional file 2: Figure S1). Because data from this and other studies show that glucocorticoids down-regulate *Lck* expression in thymocytes, we can conclude that this effect is due to heterodimerization of activated GR to transcription factors and that the GRE seems to be inactive in these cells. On the contrary, the up-regulation found in splenocytes after treatment for 6 h in vivo may be due to the GRE.

Investigation of the *Txk* promoter revealed three and two very well-conserved GRE and nGRE motifs, respectively (Additional file 1: Table S1 and Additional file 2: Figure S1). These data suggest that *Txk* expression is strictly controlled by glucocorticoids and that differences in glucocorticoid-dependent *Txk* modulation between thymocytes and T lymphocytes from peripheral organs may reflect the balance between GRE- and nGRE-mediated regulation.

Glucocorticoids affect peripheral immune responses by inhibiting T cell immunity at several stages of the activation cascade. Because the thymic epithelium produces steroids, it is reasonable to hypothesize that endogenous glucocorticoids also play a role in controlling T-cell development [33,34]. Brewer and colleagues showed that apoptosis induced by TCR triggering is mediated by glucocorticoids in murine thymocytes. Reports have also described glucocorticoid-mediated transcriptional regulation of TCR complex proteins [21,35,36], as well as modulation of TCR function and signaling transduction [37-40]. In particular, glucocorticoid treatment has been shown to modulate kinase activity in activated T cells [41]. As both peripheral responsiveness and thymic differentiation appear to be regulated by the quantity and quality of intracellular signals triggered by TCR activation, the interference of glucocorticoids on the expression of kinases downstream of the TCR may contribute to the effect of endogenous and pharmacological glucocorticoids on T cells.

Our study demonstrates for the first time that both Tec kinases are up-regulated in splenocytes and lymphocytes. However, *Itk* up-regulation was more rapid and quantitatively relevant considering that the basal levels of *Itk* expression are higher than that of *Txk* in mature lymphocytes [12]. Several findings suggest that Itk activity favors differentiation towards the Th2 phenotype [14-16,42]. The various effects of glucocorticoids on T cell differentiation in the different experimental models and human diseases depend on several factors; however, data suggest that glucocorticoids favor Th2 and Treg differentiation of CD4^+^ T cells [5,7,8]. In this context, it is likely that dexamethasone-induced *Itk* up-regulation is at least partly responsible for the Th2-polarizing effects of glucocorticoids. Moreover, Itk seems to favor Th17-induced T regulatory cell (iTreg) polarization [43] whereas glucocorticoids enhance the Th17/Th1 imbalance in patients with systemic lupus erythematosus [44].

## Conclusions

Modulation of TCR signaling by glucocorticoids is known to involve several mechanisms, including regulation of TCR complex subunit expression and kinase activity. In this study, we show that *Itk*, *Txk*, and *Lck* expression is modulated by dexamethasone in thymocytes and mature lymphocytes, demonstrating another mechanism by which glucocorticoids modulate T-cell activation. Furthermore, we reveal that dexamethasone-driven *Itk* up-regulation plays a protective role in glucocorticoid-induced thymocyte apoptosis. Dexamethasone was also found to induce differential expression of *Itk* and *Txk* in mature lymphocytes, possibly favoring T-cell polarization. Future studies are required to address the actual in vivo relevance of such a mechanism.

## Abbreviations

Ab: Antibody; Itk: IL2 inducible T cell kinase; Itk^-/-^: Itk knock-out; Lck: Lymphocyte protein tyrosine kinase; RACE: Rapid amplification of cDNA ends; RPA: RNAse protection assay; TCR: T-cell receptor; Th: T helper; Txk: Txk tyrosine kinase; Txk^-/-^: Txk knock-out.

## Competing interests

The authors declare that they have no competing interest.

## Authors’ contributions

GMP conducted PCR, RPA, and in vivo experiments, prepared figures, and contributed to the writing of the manuscript; KF conducted PCR, RPA, western blotting, apoptosis, and in vivo experiments; PM participated in research design, conducted PCR experiments, and performed data analysis; SR conducted RPA and in vivo experiments; LC conducted real-time PCR experiments; GM participated in research design and contributed to the writing of the manuscript; EM conducted in vivo experiments and contributed to the writing of the manuscript; OB conducted RPA and western blotting experiments; SB performed in silico analysis of promoters and conducted apoptosis and in vivo experiments; GN participated in research design, performed data analysis, and wrote the manuscript; CR participated in research design and contributed to the writing of the manuscript. All authors read and approved the final manuscript.

## Pre-publication history

The pre-publication history for this paper can be accessed here:

http://www.biomedcentral.com/2050-6511/15/35/prepub

## Supplementary Material

Additional file 1: Table S1 Putative glucocorticoid responsive element (GRE) and negative GRE (nGRE) sites within the murine Itk, Txk and Lck genes. Click here for file

Additional file 2: Figures S1 The putative Glucocorticoid Responsive Element (GRE) and negative GRE (nGRE) sites in murine Itk, Txk and Lck genes. Click here for file
