# Supplementary material for: Transcriptional regulation of kinases downstream of the T cell receptor: another immunomodulatory mechanism of glucocorticoids
Source: BMC Pharmacol Toxicol. 2014 Jul 3;15:35. doi: 10.1186/2050-6511-15-35 (PMC4105561; doi:10.1186/2050-6511-15-35)
Supplement: Additional file 1: Table S1 — Putative glucocorticoid responsive element (GRE) and negative GRE (nGRE) sites within the murine Itk, Txk and Lck genes. [file 2050-6511-15-35-S1.pdf]

Table s1. Putative glucocorticoid responsive element (GRE) and negative GRE (nGRE) sites within the murine Itk, Txk and Lck genes.

	NCBI ID					
**Gene name**	**DNA (gene)**	**Isoform**	**Element**	**Position**	**Strand**	**Matrix similarity**[$]
IL2 inducible T cell kinase (**Itk**)	16428	NM_001281965.1	nGRE	Exon 1<br>+119 to +133	+	0.870
			GRE	Exon 9<br>+32 to +50	-	0.900
Lymphocyte protein tyrosine kinase (**Lck**)	16818	NM_010693.3	GRE	Intron 4<br>+46 to +61	-	0.931
TXK tyrosine kinase (**Txk**)	22165	NM_001122754.2	GRE	Proximal Promoter<br>-66 to -46	-	0.884
			GRE	Intron 1<br>+15917 to +15932	-	0.894
			nGRE	Intron 1<br>+16120 to +16133	-	0.877
			GRE	Exon 2<br>+9 to +24	+	0.922
			nGRE	Exon 11<br>+105 to +118	+	0.892

$ Matrix similarity is evaluated through an algorithm comparing matrix of the element (the different GREs and nGREs) with the sequence present in the gene. A perfect match to the matrix gets a score of 1 (each sequence position corresponds to the highest conserved nucleotide at the position in the matrix).
