# Supplementary material for: Transcriptional regulation of kinases downstream of the T cell receptor: another immunomodulatory mechanism of glucocorticoids
Source: BMC Pharmacol Toxicol. 2014 Jul 3;15:35. doi: 10.1186/2050-6511-15-35 (PMC4105561; doi:10.1186/2050-6511-15-35)
Supplement: Additional file 2: Figures S1 — The putative Glucocorticoid Responsive Element (GRE) and negative GRE (nGRE) sites in murine Itk, Txk and Lck genes. [file 2050-6511-15-35-S2.pdf]

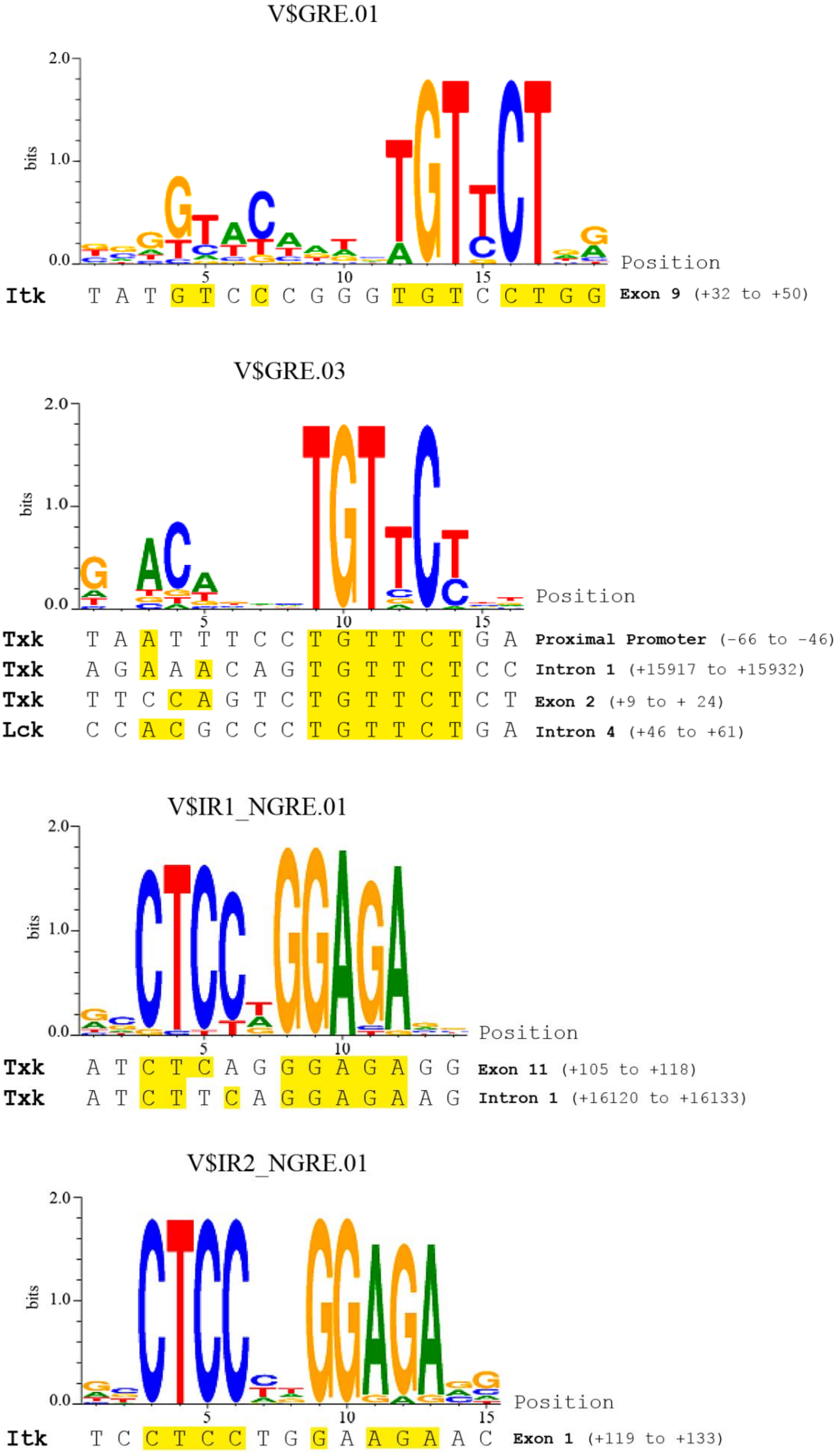

Figure s1. The putative glucocorticoid responsive element (GRE) and negative GRE (nGRE) sites found within the murine Itk, Txk and Lck genes are reported and compared to the matrix of the element. Nucleotides matching with the matrix are highlighted. Position of the putative GRE or nGRE within the gene is also reported.
